# Comparison of blood culture contamination rates with standard practice versus two blood diversion devices at a single institution

**DOI:** 10.1128/jcm.00085-26

**Published:** 2026-06-29

**Authors:** Casey Vieni, Michele Legried, Jill Mainella, Kelly Bute, Linda Backus, Priya Sampathkumar, Audrey N. Schuetz, Robin Patel, Brad S. Karon

**Affiliations:** 1 Division of Clinical Microbiology, Department of Laboratory Medicine and Pathology, Mayo Clinic 195112, Rochester, Minnesota, USA; 2 Division of Clinical Core Laboratory Services, Department of Laboratory Medicine and Pathology, Mayo Clinic 195112, Rochester, Minnesota, USA; 3 Division of Public Health, Infectious Diseases, and Occupational Medicine, Department of Medicine, Mayo Clinic 6915, Rochester, Minnesota, USA; Endeavor Health, Evanston, Illinois, USA

**Keywords:** diagnostics, culture contamination, blood cultures

## Abstract

Blood culture contamination can lead to false-positive blood culture results, potentially contributing to increased costs and unneeded antibiotic utilization. Initial specimen diversion devices (ISDD) have been proposed to reduce blood culture contamination rates. A single-center, observational study conducted at Mayo Clinic Rochester compared blood culture contamination rates using Steripath or Kurin ISDDs versus standard blood culture collection protocols. Over 255 phlebotomists were trained in the use of the ISDDs, with 31,215 blood cultures, 410 of which were contaminated, collected from adults in the emergency department and inpatient wards. Baseline contamination rates were 1.29%–2.53% with standard collection methods. The Steripath ISDD with direct-to-media inoculation of blood culture bottles (Steripath-DTM) reduced blood culture contamination compared to standard practice (0.34% vs. 1.43%, respectively, *P* < 0.001). Follow-up studies with the alternative Steripath configuration with an integrated syringe (Steripath-syringe), and then the Kurin ISDD with syringe collection (Kurin-syringe), did not meet the primary endpoint of <0.5% contamination (Steripath-syringe: 1.28%; Kurin-syringe: 1.76%). All three ISDD configurations failed to meet our goal of >75% utilization, with an average measured utilization of 26% for the Steripath-DTM, 26.4% for the Steripath ISDD (combined DTM and syringe) in a follow-up study, and 41.5% in the Kurin study. Additionally, a survey of phlebotomists revealed low staff satisfaction with both Steripath ISDD configurations. Staff reported multiple ergonomic barriers limiting the Steripath ISDD’s utility, which precluded implementation of the Steripath-DTM in routine clinical practice.

**IMPORTANCE:**

We conducted a very large, real-world study of two blood diversion devices to determine whether either would meet goals for reduced blood culture contamination and a high rate of utilization by phlebotomists.

## INTRODUCTION

Bloodstream infections and sepsis lead to an estimated 200,000 deaths annually in the United States (1, 2) and are associated with ~14% to 38% estimated mortality (3, 4). Blood cultures are the standard method for diagnosing bloodstream infections and one of the most ubiquitous and important tests performed in the clinical laboratory (5). False-positive blood culture results due to contamination may lead to patients receiving unnecessary antibiotics.

Several variables influence the performance and the cost of blood cultures, including the method and site of collection, type of media used, use of skin preparation prior to phlebotomy, and the number of cultures collected (6–11). The volume of blood assayed plays a key role in the likelihood of detecting bacteremia (12–16). Part of the cost associated with detecting bloodstream infections is due to the potential for false-positive detection of bacterial contaminants. False-positive blood cultures result in greater resource utilization through unneeded hospital admissions, unneeded additional laboratory tests and/or imaging, and unnecessary prescription of antibiotics (17–19), though rapid diagnostics applied to positive blood culture bottles have mitigated this (20–22). Laboratories should achieve contamination rates <3%, with the Clinical & Laboratory Standards Institute (CLSI) recommending a target contamination rate of ≤1% when best practices are followed (23–26). A 1998 College of American Pathologists (CAP) Q-probes quality assurance study demonstrated blood culture contamination rates varying from <1.0% to >5.0% (27).

Several techniques have been explored to reduce the rate of blood culture contamination (23). Blood culture contamination is reduced when using dedicated phlebotomy teams versus non-phlebotomy health-care staff, through education-based interventions and departmental report cards, and when blood collection is performed from a peripheral site versus a newly inserted intravenous catheter (28–33). Alternative skin antiseptic formulations used during the phlebotomy procedure have not been shown to be a large factor in decreasing blood culture contamination rates (6). Use of an initial specimen diversion device (ISDD) may potentially reduce blood culture contamination. ISDDs are pre-assembled, sterile blood culture systems designed to divert initial blood flow (from 0.2 to 2.0 mL depending upon the device) prior to culture bottle inoculation. As skin fragments can be recovered from injection needles, diversion of the initial specimen that contains skin fragments that harbor bacteria may reduce contamination (34, 35). ISDDs were shown to decrease contamination rates in an inpatient setting and in the emergency department of various institutions (36–38). A recent meta-analysis showed that the use of diversion devices reduced blood culture contamination without reducing detection of true infection (39). Collecting blood cultures is a technically challenging procedure to which the use of ISDDs may add additional complexity.

Here, an assessment of two ISDDs, Steripath and Kurin, on the blood culture contamination rates at Mayo Clinic, Rochester, Minnesota, is presented. Contamination rates, the distribution of the recovered species, and phlebotomist utilization rates and satisfaction with the devices are described.

## MATERIALS AND METHODS

### Study details

A single-center, observational study was conducted at Mayo Clinic, Rochester, Minnesota, to compare blood culture contamination using the Steripath (Magnolia Medical Technologies) and Kurin Lock (Kurin, Inc.) ISDD (Fig. S1) with standard blood culture collection protocols.

### Phlebotomy protocol

The study was performed among adult inpatients (>18 years old) with an order for a peripheral blood culture at Mayo Clinic Hospital from February 2021 to November 2022. Venipuncture was performed by trained phlebotomists; over 255 phlebotomists were trained to use the devices. Both 21- and 23-gauge needles were available for the Steripath and Kurin ISDDs, with the needle gauge used based on phlebotomist preference. Prior to venipuncture, 2% chlorhexidine gluconate and 70% isopropyl alcohol with 30 seconds of air-dry time were used to disinfect the skin. Approximately 60 mL total of blood, per patient, was collected across two blood culture draws, with ~30 mL per draw distributed to one anaerobic (Bactec Lytic Anaerobic/F) and two aerobic (Bactec Plus Aerobic/F) culture bottles, as per institutional guidelines (16). The standard practice is to collect 30 mL of blood into one or more syringes and then transfer it into blood culture bottles. The paired culture draws are typically obtained from opposite arms. Standard aseptic practices were followed for both ISDDs. Blood culture vial tops were cleaned with 70% isopropyl alcohol pads before inoculation. Blood cultures were incubated for 5 days on a Bactec FX instrument system at 35°C and, if positive, contents were subcultured and organisms identified by standard techniques. In addition, rapid diagnostics, including the BioFire FilmArray Blood Culture Identification 2 (BCID2; bioMérieux, Marcy-l'Étoile, France) and Accelerate Pheno (Becton Dickinson, New Jersey, USA), were performed on gram-positive bacteria/yeast and gram-negative bacteria, respectively. Rapid matrix-assisted laser desorption/ionization-time of flight (MALDI-TOF) mass spectrometry (Bruker Scientific, Billerica, MA; library: Mayo Clinic Rochester Library, Bruker BDAL Database, and Security-Relevant (SR) Library) was performed for identification of any organisms not identified using the aforementioned rapid diagnostic tests.

Single draws positive for coagulase-negative staphylococci (aside from *Staphylococcus lugdunensis*), diphtheroids, *Bacillus* species, *Micrococcus* species, *Cutibacterium* species, or viridans streptococci were classified as contaminants (25, 27). In instances where these same organisms were isolated from two or more blood culture sets, they were classified as possible pathogens instead of contaminants.

### Steripath ISDD

First, a baseline peripheral blood culture contamination rate was established for 8 weeks using standard practice prior to the first ISDD pilot. Next, an 8-week study (Steripath-DTM study) was performed in which the blood culture contamination rates for the Steripath ISDD were separately monitored and compared to contamination rates for any phlebotomy procedures using standard practice. Specimens were collected with direct-to-media inoculation of blood culture bottles (Steripath-DTM) with the Steripath Gen2 and the integrated transfer adapter direct-to-media configuration (Magnolia Medical Technologies; Catalog #2700-21-EN and 2700-23-EN for 21- and 23-gauge formulations, respectively) according to the manufacturer’s instructions. This collection method avoids syringe transfer of blood into blood culture bottles. Success for the pilot was predefined based on manufacturer recommendations as a contamination rate of <0.5%, ≥50% reduction from baseline contamination rates, and >75% utilization of the device. Device utilization was defined as the number of cultures collected with the ISDD divided by the total number of cultures collected during a particular study period. All phlebotomists collecting adult blood cultures in the emergency department (ED) or any inpatient unit were trained on the use of the Steripath-DTM device. Use of the ISDD was encouraged, but the phlebotomist could use standard practice according to their judgment. Phlebotomists were allowed to collect specimens using standard practice for difficult blood draws or if the phlebotomist anticipated a low blood volume. Upon review of the first 8 weeks of the study, the measured utilization rate was low (~20%–30%); the study was extended an additional 10 weeks (total of 18 weeks) to better assess the ISDD following additional education and reminders to phlebotomy staff to use the ISDD when possible.

Following the completion of this initial study, a second study (combined Steripath study) was implemented in which the ISDD could either be used via Steripath-DTM or with the integrated 10 mL syringe configurations (Steripath-syringe; Catalog #2720-21-EN and 2720-23-EN for the 21- and 23-gauge formulations, respectively), an alternative collection method. Since syringe collection is common at our institution, the syringe collection option was added as an option for Steripath collections to potentially increase utilization. This study was concluded after 9 weeks.

### Kurin ISDD

Following completion of the Steripath studies, a third study (Kurin-syringe study) was conducted using the Kurin Lock ISDD with syringe collection (Kurin-syringe; Catalog #M21221 and M21223 for the 21- and 23-gauge formulations, respectively). Based upon experience (i.e., low utilization and unfavorable user assessment) with the Steripath-DTM in the first study, only syringe collection was used with the Kurin ISDD. Kurin-syringe utilization and contamination rates were monitored weekly, and use of the ISDD was encouraged for all peripheral collections. As the contamination rates and utilization with the device did not meet the respective target goals of <0.5% contamination and >75% utilization, respectively, the third study was terminated after 5 weeks.

### Data analysis

Data analysis was completed using R version 4.3.1 (40, 41). Results were analyzed per individual blood culture draw rather than per set of blood cultures. During each study, the method of collection (standard practice versus ISSD) was documented by phlebotomy at collection by changing the collection code in the laboratory information system (LIS), with standard collection being the default selection. Similarly, in the combined Steripath study, use of the Steripath-DTM versus Steripath-syringe was documented in the LIS, with standard collection being the default selection. Fisher’s exact test was used to compare proportions of blood cultures that were contaminated, as well as those yielding pathogens.

### Survey design

Upon completion of each respective Steripath study, an anonymous 11-question post-study survey (Table S1) was distributed to phlebotomists who used the device to gather qualitative information about user experience. The first survey, sent after 18 weeks, evaluated the Steripath-DTM. The second survey was sent upon completion of the second 9-week study and only evaluated the targeted phlebotomists’ experience with the Steripath-syringe collection process. Survey questions included free-text responses, as well as questions measured via the Likert scale. A survey was not conducted for the Kurin ISDD due to the early termination of this arm of the study.

## RESULTS

### Standard practice contamination rates

A total of 31,215 blood culture draws were included in this study (Table 1), with 410 contaminated blood cultures (mean contamination rate of 1.31% across all draws). Prior to implementation of ISDDs, 6,747 specimens were collected over a 2-month period, with 88 contaminated draws, for a baseline contamination rate of 1.30%. Culture positivity rates during this period were 8.4% and 9.9% compared to average monthly blood culture positivity rates of 7%–10% observed in the laboratory. Across all three studies, 14,491 draws were collected via standard practice (i.e., phlebotomists used standard practice rather than an ISDD), of which 202 (1.39%) were contaminated.

**TABLE 1:** Total number of blood culture specimens, contaminated blood draws, and percent utilization of the Steripath and Kurin ISDDs with integrated DTM configurations or integrated syringe configurations compared to standard practice

Study		Diversion device			Standard practice
		Steripath-DTM^*a*^	Steripath-syringe	Kurin-syringe	
Baseline	Length of study	8 weeks			
	Total draws				6,747
	Contaminated blood draws				87
	Percent contaminated blood draws (%)				1.29
Steripath-DTM study	Length of study	19 weeks			
	Total draws	3,482			9766
	Contaminated blood draws	12			140
	Percent contaminated blood draws (%)	0.34			1.43
	Percent utilized (%)	26.3			
Combined Steripath study	Length of study	8 weeks			
	Total draws	1,490	1,168		4,725
	Contaminated blood draws	7	14		59
	Percent contaminated blood draws (%)	0.47	1.20		1.25
	Percent utilized (%)	14.8	11.6		
Kurin-syringe study	Length of study	5 weeks			
	Total draws			1,591	2,246
	Contaminated blood draws			27	34
	Percent contaminated blood draws (%)			1.70	1.51
	Percent utilized (%)			41.5	

^*a*^ DTM, direct to media.

### Steripath-DTM

**Fig 1:**
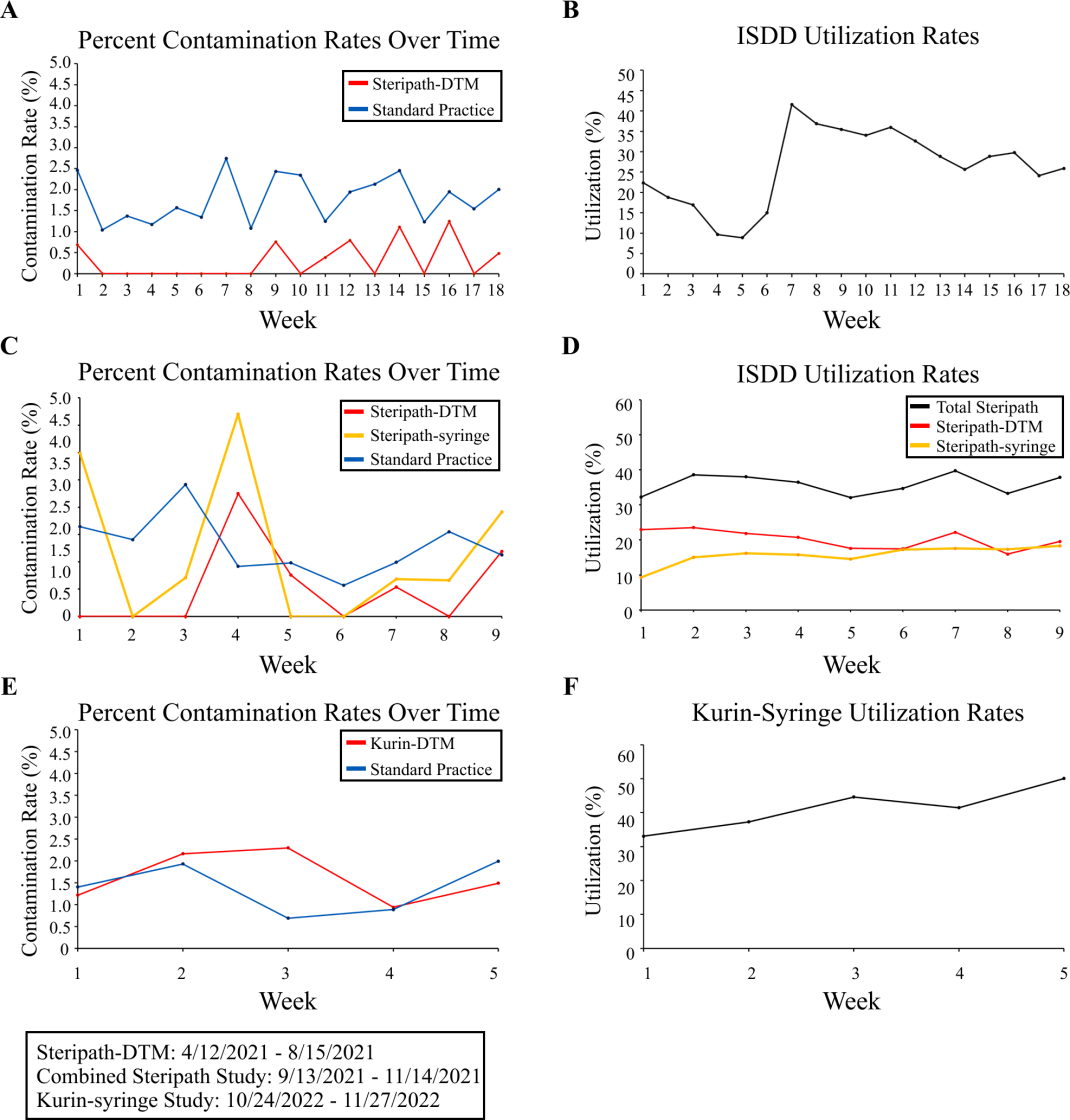
Initial specimen diversion device (ISDD) percent contamination rates and utilization over time across the various phases of this study. (**A**) Contamination rates and (**B**) device utilization for the initial study of the Steripath with the integrated transfer adapter direct-to-media configuration (Steripath-DTM). (**C**) Contamination rates and (**D**) device utilization during the combined Steripath study comparing the Steripath device with the integrated syringe configuration (Steripath-syringe; yellow) and the Steripath-DTM (red) collection method. (**E**) Contamination rates and (**F**) utilization of the Kurin Lock ISDD device. Contamination rates using standard practice phlebotomy are shown in blue.

First, during the initial 8 weeks of the study assessing the Steripath-DTM ISDD, at least 1,199 draws were collected with the Steripath ISDD versus 4,452 collected per standard practice (average utilization rate of 26.9%; Fig. 1A and B). Over the first 8 weeks, one (0.08%) blood culture collected using the Steripath-DTM was contaminated during this study versus 64 (1.43%) when using standard phlebotomy practice. Contamination rates following implementation of the diversion device were lower than those prior to implementation of the Steripath ISDD (*P* < 0.001).

ISDD utilization was tracked weekly, and at the 6-week time point, near the conclusion of this study, utilization was 15.3% (Fig. 1B). Therefore, the study was extended for an additional 10 weeks beyond the original 8 weeks following additional staff education and reminders. Although there was an increase in utilization to ~39% during weeks 7 and 8 following staff education and reminders, utilization trended downwards over the following 10 weeks (weeks 9–18) with an average utilization of ~30%. The average weekly utilization was 26.1% across the entire 18-week study (Fig. 1B). This was likely due to staff discomfort with the device (see *Survey Results* subsection). Across the full 18 weeks of this study, the contamination rate for the Steripath-DTM (0.34%) was lower than with standard collection methods (1.43%) (Table 1; Table S1; *P* < 0.001). Across the 18 weeks of the Steripath-DTM study, the monthly blood culture positivity rate for specimens collected with the Steripath-DTM ranged from 6.6% to 9.5%, suggesting no change in pathogen detection when using the Steripath-DTM.

### Steripath-DTM versus Steripath-syringe

A second study comparing Steripath-DTM, Steripath-syringe, and standard collection method included at least 2,658 specimens collected via the Steripath ISDD, with 1,168 cultures collected via the Steripath-syringe, and 1,490 specimens collected via the Steripath-DTM (Table 1). We recorded 4,725 draws collected using standard practice during the combined Steripath study. The overall contamination rate for the Steripath device (Steripath-syringe and Steripath-DTM) during this study was 0.87%, with contamination rates for Steripath-syringe and Steripath-DTM of 1.28% and 0.54%, respectively (versus standard practice: 1.25%). The collection of cultures via Steripath-syringe did not result in a statistically significant difference in contamination rates (*P* = 1.0), compared to standard collection, while the use of Steripath-DTM resulted in a statistically significant difference (*P* < 0.01) in contamination. The combined (Steripath-syringe plus Steripath-DTM) contamination rate also did not differ from standard practice (*P* = 0.08). Across this study, the overall utilization of the Steripath ISDD (Steripath-syringe and Steripath-DTM) was 36.0%, with 15.8% of draws collected via Steripath-syringe and 20.2% collected via Steripath-DTM. The blood culture positivity rates during the combined Steripath study ranged from 8.6% to 11.6%, again suggesting no change in pathogen detection using the Steripath ISDD.

Across both the first 18-week study and the second 9-week study, at least 4,972 blood culture draws were collected using the Steripath-DTM with a contamination rate of 0.40%. Overall utilization of the Steripath-DTM configuration across the Steripath-DTM (first) study and combined Steripath (second) study was 24.1%.

### Kurin ISDD

A third study was conducted using the Kurin Lock ISDD (Kurin-syringe). In total, 3,837 draws were collected during this study, with at least 1,591 draws collected using the Kurin-syringe and at most 2,246 draws using standard practice. A total of 27 contaminants were recovered from specimens collected using the Kurin-syringe (1.70%), while 31 contaminants (1.38%) were recovered from the specimens collected using standard practice. There was no difference (*P* = 0.70) in contamination rates from specimens collected using the Kurin ISDD versus standard collection procedures. During the only complete month of the Kurin study, the percentage of positive blood cultures was 7.3%. Overall, utilization of the Kurin ISDD was 41.5%.

The Kurin ISDD did not meet the goal of a contamination rate <0.5% and utilization >75%. Additionally, informal, real-time queries of staff showed low satisfaction with the Kurin device. Therefore, the Kurin ISDD study was suspended after 5 weeks, and a follow-up survey was not conducted.

### Contaminating organisms

An array of organisms was identified as contaminants during this study (Table S2). Coagulase-negative staphylococci were the most recovered contaminants across all ISDDs, with *Staphylococcus epidermidis* being the most commonly recovered species, consistent with the literature (42).

### Device utilization and contamination rates by hospital ward

During the study periods, approximately 5%, 10%–20%, and 65%–75% of blood cultures were collected from the emergency department (ED), intensive care units (ICU), and general inpatient wards. Across all three study periods, utilization of the respective devices was comparable across different hospital wards with a weekly average of ~4%–6% of ISDD draws occurring in the ED and a weekly average of ~8%–16% of ISDD draws occurring in the ICU across all study periods (given by the number of specimens collected with an ISDD per ward divided by the total number of specimens collected with an ISDD).

Weekly contamination rates stratified by hospital ward were highest in the general wards, then the ICU, and lowest in the ED across all study periods. In the Steripath-DTM study, average weekly contamination rates for standard practice were 1.8% (ICU) and 1.8% (ED) compared to 2.4% (ICU) and 0% (ED) for the Steripath-DTM. Average weekly contamination rates in the combined Steripath study were 1.8% (ICU) and 1.9% (ED) for standard practice, 1.6% (ICU) and 0% (ED) for the Steripath-DTM, and 2.1% (ICU) and 4.2% (ED) for the Steripath-syringe. In the Kurin study, standard practice weekly average contamination rates were 3.3% (ICU) and 2.3% (ED) compared to 0% (ICU) and 2% (ED) with the Kurin-syringe. At our institution, a dedicated phlebotomy team collects all blood cultures (including in the ED), which has been shown to reduce blood culture contamination rates (30, 31, 43). Hence, the increased contamination rates for standard practice draws in the wards or ICU may reflect the increased prevalence of draws through intravascular lines, while blood cultures collected in the ED are much more likely to be through a peripheral draw.

### Survey results

**Fig 2:**
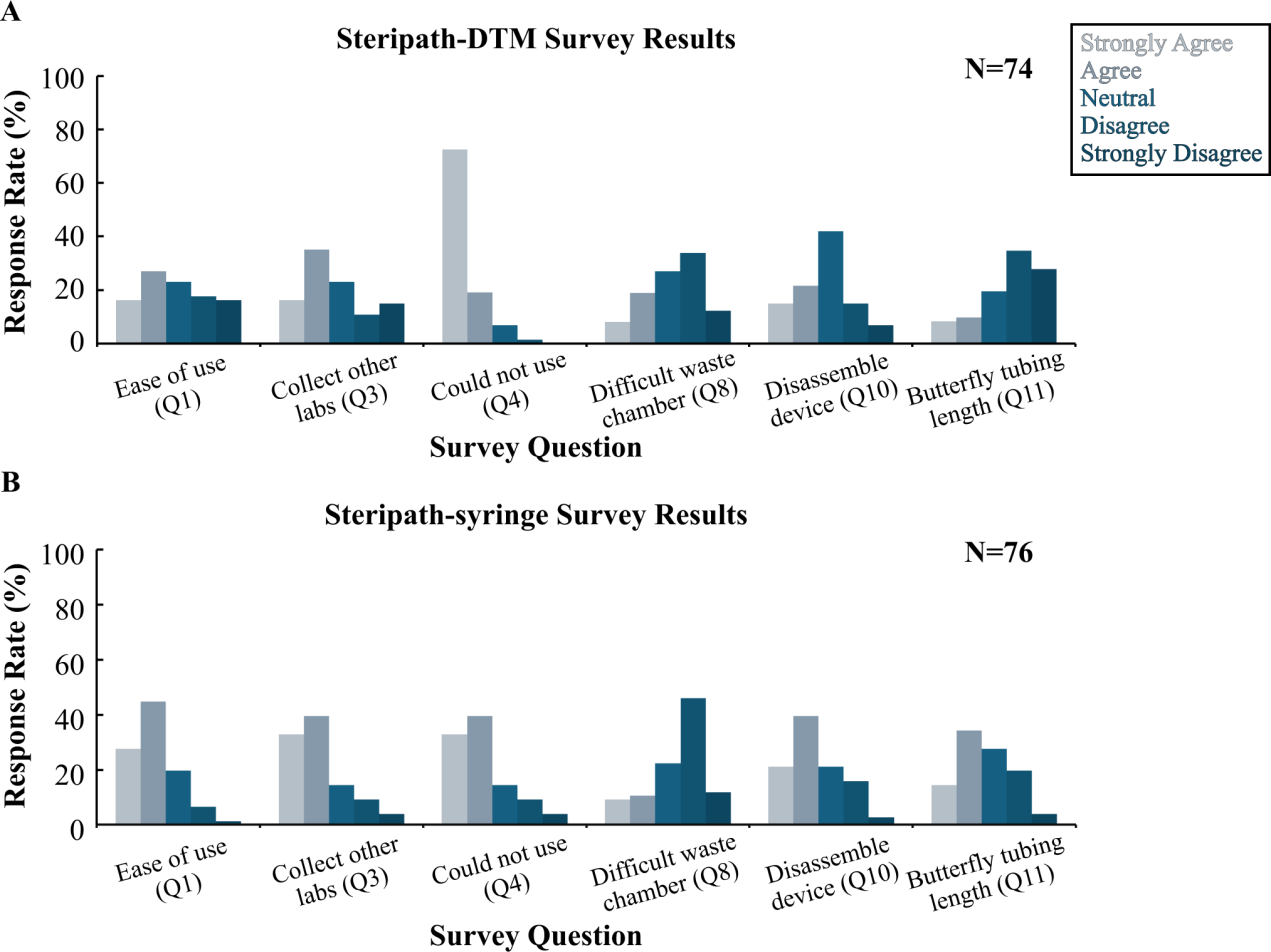
Aggregate survey responses to questions with a Likert scale response following (**A**) the study of the Steripath initial specimen diversion device (ISDD) with an integrated transfer adapter direct-to-media configuration (Steripath-DTM) and (**B**) the Steripath ISDD with an integrated syringe configuration (Steripath-syringe). Survey responses correspond to questions 1, 3, 4, 8, 10, and 11 (see Table S1 for complete question text).

In total, 74 of 215 surveys sent to the phlebotomy team to gather qualitative information about their experience with the Steripath-DTM were completed (Tables S3 and S4; Fig. 2). Only 43.2% (32/74) of phlebotomists stated that the Steripath-DTM was easy to incorporate into routine blood draws. Similarly, 76 of 215 surveys sent to the phlebotomy team to evaluate the Steripath-syringe were completed (Tables S3 and S5; Fig. 2C and D). 72.4% (55/76) of phlebotomists stated that the Steripath-syringe could be easily incorporated into routine blood draws and could be used to collect other blood for other laboratory tests without difficulty. The diversion aspect of either configuration of the ISDD was generally not difficult to use, with phlebotomists mostly disagreeing that it was difficult to activate the waste chamber, 45.9% (34/74) with the Steripath-DTM and 57.9% (44/76) with the Steripath-syringe. However, 90.5% (67/74) and 72.4% (55/76) of phlebotomists agreed that the Steripath-DTM and Steripath-syringe configurations could not be used with certain adult patient populations, respectively.

While training with the device was noted as adequate for both configurations (total yes: 125/145 [86.2%] vs. no: 13/145 [9.0%]), free-text answers for “Did you try to use the Steripath syringe device for every blood culture order? If No, why not?” (Q6) were variable. Specifically, for the Steripath-DTM, phlebotomists occasionally remarked that “you must be level with the bottle” and “you need a flat surface” to get an accurate volume. As “there is not a flat surface available in certain areas” (as noted by one phlebotomist), several phlebotomists reported that the Steripath-DTM was “hard to see” and they had to “kneel on [the] floor to see the line of blood.” This led to feelings that the “bottles [were] marked incorrectly” with “inconsistency in the broth lines” and that it was “impossible to…see correctly and prevent over or under filling.” The “tubing [was] too short for adjustment” (noted by one phlebotomist), and together, these issues contributed to occupational hazards, such as “backaches even with [the] bed raised,” as phlebotomists were required to “contort [their bodies] into weird positions.” Several responses mentioned difficulty with crouching while using the device; this led to ergonomic challenges for tall phlebotomists, as the patient’s bed could not be raised high enough to accommodate the use of the device.

Specifically, for the Steripath-syringe, phlebotomists found that switching out syringes was challenging with the ISDD device. The syringe device “seal[ed] to[o] well and is hard to twist off.” As the “butterfly did not unscrew,” there was “no way to stabilize” the needle, and the needles “fall out of [the] arm.” Phlebotomists reported that they could not easily switch out syringes and disassemble the device, with only 14/76 (18.4%) agreeing or strongly agreeing that it was easy to swap out syringes to obtain a full volume blood culture collection.

Both the Steripath-DTM and Steripath-syringe were “very bulky” compared to standard collection, and the size of the devices was challenging. This led to the device taking up more physical space on transport carts, contributing to inventory challenges and mishandling issues, such as “feel[ing] easier to accidentally pull the needle out” and having “not enough hands to do everything required.” This was particularly true in busy rooms, emergency situations, or if the patient had behavioral issues and/or could not hold still. Several phlebotomists described avoiding the device in code situations, intensive care unit (ICU) patients, and anxious or combative patients.

Particularly for the Steripath-DTM, phlebotomists felt that the size of the bottle vacuum on the device limited its use in patients with small-caliber, fragile veins, and some reported switching to standard protocols after suspecting that use of the device may collapse a patient’s veins. Hence, this device was of limited use in patients with “swollen arms, bruises, or poor veins.” When assessing vasculature for a blood draw, some phlebotomists reported avoiding using the Steripath if the “chances of getting 30 mL were slim,” the patient had “hematomas or swollen arms,” or if their “professional judgment said the device would damage the only vein available.”

## DISCUSSION

The Steripath and Kurin ISDDs may serve as potential methods to reduce blood culture contamination rates in ED and inpatient settings. The devices are pre-assembled, sterile systems that divert the initial ~2.0 mL of blood (Steripath) or 0.2 mL of blood (Kurin) containing skin fragments prior to blood culture inoculation, without reducing the detection of true infection (39). The range of contamination rates using standard collection procedures was 1.29%–1.43% over the course of this study, compared to 0.40% with Steripath-DTM (4,972 total collections), 1.28% with Steripath-syringe (1,168 total collections), and 1.76% with Kurin-syringe (1,591 total collections). The goal contamination rate of <0.5% was only met with Steripath-DTM, which the phlebotomy staff unfortunately found difficult to use, leading to low device utilization (weekly average 26.1%). The rate of positive blood cultures collected with ISDD devices did not vary from the range of positivity rates experienced by the laboratory with routine collection.

Studies evaluating the impact of ISDDs are heterogeneous with respect to technique, research design, specific ISDD devices used, clinical environment, and patient population (44). The Steripath has been reported to reduce contamination rates in one randomized controlled trial of 1,016 cultures (37), three nonrandomized prospective studies (36, 38, 45), and two quasi-experimental studies (46, 47), though contamination rates of <1% were inconsistently achieved across the reported studies. The Kurin device has been evaluated in several quasi-experimental studies with numerical reduction in contamination rates, although only one study met the goal of <0.5% contamination (48–50). Alternatively, an “open technique” in which the initial specimen diversion occurs without a dedicated device may offer a reduction in contamination rates without additional costs of a commercial device (35, 51). For example, in one study of 810 cultures, all blood cultures in the intervention arm were collected *after* blood was collected for routine chemistries in a sodium-heparin tube (hence diverting the first ~6–10 mL of blood), leading to a reduction in contamination rates from 5% to 1.7% (51). Similarly, another report of 3,733 cultures in which the first 3.0 mL of blood was diverted into a sterile vacutainer before inoculating cultures yielded a reduction in aerobic bacterial contamination rates from 2.8% to 1.4%, but no difference in anaerobic bacterial contamination rates (35). Across studies exploring ISDD use, reported users included phlebotomists, ED and inpatient nurses, nursing assistants, training service members, and resident physicians (37–39, 46, 52).

Direct comparisons of ISDDs are rarely reported in the literature (39). One study by Arenas et al. compared 4,030 specimens collected exclusively in the ED by nurses and emergency technician nurse assistants using early versions of the Steripath (1–2 mL diverted; 664 specimens) and Kurin (0.15 mL diverted; 1,312 specimens) devices. The authors reported decreases in contamination rates (0.0% and 0.3% for the Steripath and Kurin ISDDs, respectively, versus 2.2%–5.2% without ISDD) (52). Similarly, phlebotomists are rarely queried about their experience with the device, with only one study reporting evaluation surveys (8 responses of 11 phlebotomists queried) for the Steripath-DTM. The device was considered easy to use because of direct culture inoculation without the need to transfer blood from a syringe to blood culture bottles. However, phlebotomists avoided using the ISDD for difficult clinical environments or patients perceived to have small or fragile veins (36). The large amount of survey data is a strength of the current study.

This study has several limitations. Foremost, this was a real-time, prospective quality improvement project conducted over approximately ~1.5 years, with >255 phlebotomists trained on the use of the devices. Neither a specific group of phlebotomists nor a specific patient population was targeted for this study. Although device education and re-education were periodically performed, a different cohort of phlebotomists, with different levels of experience, likely completed each phase of the trial, as Mayo Clinic phlebotomist turnover rate is ~30%–40% (similar to reported 3-year turnover rates of 24.9% from a CAP survey of 20 institutions) (53). Additionally, given the overall length of this study (~4/2021 to ~11/2022), study fatigue may have contributed to the worse performance of the Kurin ISDD. Notably, the Kurin device performed worse than standard practice, though this difference was not statistically significant. Nevertheless, this study provides a real-world assessment of ISDD utilization.

Measured utilization of both the Steripath and Kurin devices was ~20%–40%, well below the target utilization of >75%. This may have been affected by the phlebotomists’ discomfort with the ISDD device. It is also likely that the ISDDs were not accurately coded in the LIS (and counted in the study) due to failure to change the default collection code and, thus, were inadvertently counted as blood draws per standard protocol. Review of our inventory during the study periods revealed that during the Steripath-DTM study, approximately 3,090 Steripath-DTM devices were used from 4/12/21 to 7/25/21 compared to the 2,849 Steripath-DTM devices that were documented (92.2%). Similarly, inventory records from 9/13/21 to 10/17/21 indicate 713 Steripath-syringe devices and 997 Steripath-DTM devices used. In contrast, phlebotomists only recorded using the Steripath-syringe in 574 draws (63.0%) and the Steripath-DTM device in 856 draws (85.9%). We estimate that 1,888 Kurin devices were used from 10/24/22 to 11/20/24, with phlebotomists documenting only 1,189 (63.0%) draws using the Kurin-syringe. This would have led to an underestimation of the number of collections that used an ISDD and, therefore, underestimated the utilization rates. While the Steripath-DTM collection met the contamination goals, culture collection using either Steripath-syringe or Kurin-syringe did not meet contamination goals. This may indicate that any benefit of the ISDD was offset by the use of the syringe to transfer blood into culture bottles. Staff found the Steripath-DTM difficult to use, as reflected in lower utilization of Steripath-DTM compared to syringe devices. Staff found the ISDDs bulky and difficult to use in certain patient populations, busy clinical environments, and emergency situations. It is possible that newer designs of these devices may alleviate some of these issues. If staff utilize the ISDD with DTM inoculation, institutions may be able to meet the defined goal of contamination rates < 0.5% and ≥50% reduction in current contamination rates. However, it was ultimately concluded that with a DTM device, >75% utilization among phlebotomy staff was unlikely to be possible in our practice.

## SUPPLEMENTAL MATERIAL

Figure S1 jcm.00085-26-s0001.png Photos of ISDD devices.

Table S1 jcm.00085-26-s0002.pdf Contamination rates over time.

Table S2 jcm.00085-26-s0003.pdf Contaminating organisms.

Table S3 jcm.00085-26-s0004.pdf Survey questions.

Table S4 jcm.00085-26-s0005.pdf Steripath DTM survey.

Table S5 jcm.00085-26-s0006.pdf Steripath syringe survey.

ASM does not own the copyrights to Supplemental Material that may be linked to, or accessed through, an article. The authors have granted ASM a non-exclusive, world-wide license to publish the Supplemental Material files. Please contact the corresponding author directly for reuse.
