# Supplementary material for: Comparison of blood culture contamination rates with standard practice versus two blood diversion devices at a single institution
Source: J Clin Microbiol. 2026 Jun 29;64(8):e00085-26. doi: 10.1128/jcm.00085-26 (PMC13463775; doi:10.1128/jcm.00085-26)
Supplement: Supplementary file 1 [file jcm.00085-26-s0002.pdf]

Table S1: Total blood draws and contaminated specimens observed per week for the Steripath and Kurin initial specimen diversion devices (ISDDs) compared to Mayo Clinic's standard practice.

	Diversion Device															
	Baseline		Steripath-DTM study				Combined Steripath study						Kurin-syringe study			
	Standard Practice		Steripath - DTM		Standard Practice		Steripath - DTM		Steripath - Syringe		Standard Practice		Kurin		Standard Practice	
**Week**	TD	CD	TD	CD	TD	CD	TD	CD	TD	CD	TD	CD	TD	CD	TD	CD
**Standard Practice Baseline Contamination Rates (2/1/2021 - 3/30/2021)**																
1	746	12														
2	706	9														
3	719	9														
4	663	9														
5	839	15														
6	894	13														
7	862	4														
8	922	11														
3/29-3/30	396	5														
**Steripath-DTM Study (4/12/2021 - 8/15/2021)**																
1			146	1	507	8										
2			133	0	575	6										
3			119	0	583	8										
4			73	0	683	8										
5			56	0	573	9										
6			118	0	669	9										
7			285	0	401	11										
8			269	0	461	5										
9			263	2	478	8										
10			242	0	469	11										
11			260	1	463	4										
12			252	2	521	6										
13			190	0	469	10										
14			180	2	522	7										
15			263	0	649	8										
16			241	3	569	4										
17			185	0	582	9										
18			207	1	592	9										
**Combined Steripath Study (9/13/2021 - 11/14/2021)**																
1							165	0	67	2	487	8				
2							191	0	122	0	498	7				
3							190	0	141	1	539	13				
4							178	4	135	5	545	5				
5							132	1	109	1	510	3				
6							141	0	139	0	527	3				
7							186	0	147	1	505	5				
8							139	0	151	1	581	9				
9							168	2	157	3	533	6				
**Kurin-syringe Study (10/24/2022 - 11/27/2022)**																
1													246	3	498	7
2													277	6	466	9
3													348	8	432	3
4													318	5	449	4
5													402	5	401	11

TD: Total Blood Draws; CD: Contaminated Draws; DTM: Direct to Media
