# Supplementary material for: Comparison of blood culture contamination rates with standard practice versus two blood diversion devices at a single institution
Source: J Clin Microbiol. 2026 Jun 29;64(8):e00085-26. doi: 10.1128/jcm.00085-26 (PMC13463775; doi:10.1128/jcm.00085-26)
Supplement: Supplementary file 2 [file jcm.00085-26-s0003.pdf]

Table S2: Distribution of contaminating organisms observed with use of the Steripath and Kurin initial specimen diversion devices (ISDDs) compared to Mayo Clinic's standard practice.

Contaminant Organism	Baseline Standard Practice	Diversion Device			
		Steripath-DTM (Steripath-DTM study)	Steripath-DTM (Combined Steripath study)	Steripath-syringe	Kurin-syringe
**Total Contamination (N)**	87	12	8	13	27
Bacillus cereus/thuringiensis	1	--	--	--	1
Bacillus licheniformis	1	--	--	--	--
Bacillus subtilis	--	--	--	1	--
Cogaulase negative Staphylococcus species	22	2	--	2	4
Corynebacterium amycolatum	1	1	--	1	--
Corynebacterium aurimucosum/minutissimum	--	--	--	1	1
Corynebacterium coyleae	1	--	--	--	1
Corynebacterium glucuronolyticum	--	--	--	--	1
Corynebacterium group F1	1	--	--	--	--
Corynebacterium simulans	1	--	--	--	--
Corynebacterium striatum	1	--	--	--	--
Cutibacterium acnes	1	--	1	--	--
Facklamia ignava	--	--	--	--	1
Microbacterium paraoxydans	1	--	--	--	--
Micrococcus luteus	--	--	--	--	2
Paenibacillus species	1	--	--	--	--
Parvimonas micra	--	--	--	--	1
Staphylococcus argensis/pettenkofferi	3	--	1	1	--
Staphylococcus capitis	--	1	--	--	--
Staphylococcus devriesei/haemolyticus	2		--	--	--
Staphylococcus epidermidis	40	5	5	5	10
Staphylococcus hominis	5	1	--	1	--
Staphylococcus salivarius group	1	1	--	1	1
Streptococcus bovis group	--	--	--	--	1
Streptococcus mitis group	4	1	1	--	3
DTM: Direct to media					
