# Supplementary material for: Comparison of blood culture contamination rates with standard practice versus two blood diversion devices at a single institution
Source: J Clin Microbiol. 2026 Jun 29;64(8):e00085-26. doi: 10.1128/jcm.00085-26 (PMC13463775; doi:10.1128/jcm.00085-26)
Supplement: Supplementary file 3 [file jcm.00085-26-s0004.pdf]

Table S3: Question list for the 11-question survey that was distributed to Mayo Clinic phlebotomists upon completion of the two Steripath studies.

Question #	Questions:	Queried Response
1	I was able to easily incorporate and use the Steripath Device into my draws	Likert scale
2	Please describe any difficulties in using the Steripath Device	Free text
3	I was able to use the Steripath w/syringe device not only to collect blood cultures but also other lab tests without difficulty	Likert scale
4	I found in certain patients or patient populations I could not use the Steripath w/syringe device	Likert scale
5	When unable to use the Steripath Device for certain patients or patient populations, what limited the use of the device?	Free text
6	Did you try to use the Steripath Device for every blood culture order? If No, why not?	Free text
7	What gauge did you prefer, 21g or 23g, and Why?	A, B, A&B, neither, no preference
8	It was difficult to engage the waste chamber	Likert scale
9	Did you feel you had adequate training to use the Steripath Device? If No, what would have made the training better?	Free text
10	I was able to disassemble the Steripath Device to use a syringe for POC testing	Likert scale
10*	I was able to easily swap out syringes as needed to obtain a full volume blood culture collection with the Steripath w/syringe device?	Likert scale
11	The butterfly tubing on the Steripath Device was of sufficient length to easily fill the bottles?	Likert scale
12†	Please describe any difficulties in measuring the volume of blood while filling the bottles you encountered?	Free text

*Only included in Phase II of this study
†: Only included in Phase I of this study
††: Likert scale of 1 (Strongly Disagree) to 5 (Strongly Agree)
