# Supplementary material for: Comparison of blood culture contamination rates with standard practice versus two blood diversion devices at a single institution
Source: J Clin Microbiol. 2026 Jun 29;64(8):e00085-26. doi: 10.1128/jcm.00085-26 (PMC13463775; doi:10.1128/jcm.00085-26)
Supplement: Supplementary file 4 [file jcm.00085-26-s0005.pdf]

Table S4: Aggregate survey responses from phlebotomists describing their experience with the Steripath with the transfer adapter direct-to-media configuration (Steripath-DTM).

**Tabulated Survey Responses:**
**2. Please describe any difficulties in using the Steripath Device**
a. The tubing is much too short
i. Makes it difficult to draw on a jumpy patient
ii. Difficult filling bottles and seeing volume – lots of bubbles in the sample
iii. Difficult to manage/hold butterfly in place when replacing bottles/tubes without dislodging the needle
iv. Difficult to keep the bottle level when filling
b. Difficult keeping the bottles clean and upright at the bedside when unable to use the patient tray or get the cart close enough (ICU's and General floors)
i. Awkward holding the bottle in the Steripath device while filling,
c. Often blood would stop or just trickle into the tube, even when using the method for fragile veins.
d. It requires excellent veins which is far and few between without patient population:
i. When "popping" the device to collect the waste it also blows the vein sometimes resulting in an addition, unnecessary poke.
e. There is no good way to incorporate Steripath with a difficult draw, during a trauma, resus, or traumatic situations
i. You must have a very good vein to guarantee a full draw
ii. These draws take SIGNIFICANTLY longer than regular culture draws, slowing us down quite a bit in the ED.
f. It is hard to use the waste device on the Steripath
g. Steripath can't work well with patients who are not settled no way to hold the butterfly and swap out specimen containers
i. Patients who can't sit still
ii. Patients who have anxiety
iii. Patients with fragile veins where the pressure in the veins are low hence the flow through Steripath is compromised
h. The device is not very ergonomic very uncomfortable draw positions to ensure volume is correct and bottles are upright
i. Even with raising the beds, still must bend over to see the volumes
ii. Kneeling on the floor to see how much blood in the bottles
i. Can't do minimum collections when the patient is a difficult collection resulting in additional pokes for the patient
j. Harder to perform heparin syringe draws and POC draws because you must take the thing apart
i. Device will not pull the blood so have to replace with a syringe (take apart the device)
ii. Unable to get the device apart when the collection was difficult
k. In the outpatient this device would work very well, but not in the inpatient population
l. Unsuccessful on patients with smaller veins most times causing additional pokes
m. Poor patient care when we must poke the patient's multiple times for 1 set of cultures
**5. When unable to use the Steripath Device for certain patients or patient populations, what limited the use of the device?**
a. Very small veins, fragile veins, the pressure was to much for some of them, multiple IV's
b. Difficult collections, restricted arms/limited options, hand veins, skin integrity
c. Bulky and hard to use and no visible flash to indicate vein is accessed
d. Combativeness of patients, behavioral patients, dementia, Timed situations,
e. Inability to hold everything properly and get the sample, needed an additional set of hands
f. Busy rooms and working around other services, emergency situations, patient cannot hold still
**6. Did you try to use the Steripath Device for every blood culture order? If No, why not?**
a. Difficult patient collections, small veins, damaged veins, behavioral patients
b. If the chances of getting 30mLs were slim did not use
c. Patients with big hematomas or swollen arms
d. Works better with big veins and often blows veins
e. Time constraints, combative patients, aggressive patients, patients unable to sit still, confused patients
f. When professional judgment said the device would damage the only vein available
g. When antibiotics had already been started
h. Work mostly with pediatric patients
i. Think it is inefficient and takes too much time
j. Frustrating to place the bottle vertical, mark the bottle to determine volumes, very awkward collection positions
k. Codes, Resus's, certain ICU patients
l. Not enough time, room, or veins to sustain the vacuum on the bottle
m. Device not working and requiring additional sticks for the patient
n. Room size, people in the room, anxiety, and mood of patient
**7. What gauge did you prefer, 21g or 23g, and Why?**
a. 21g, n = 52
i. better flow
ii. Because of reduced hemolysis
iii. Made collection faster
b. 23g, n = 4
i. Able to use on most patients
ii. Smaller needle
iii. More successful

iv. Harder time using the 21g

c. Both, n =9

i. Used what was best for the vein

d. Neither, n = 5

i. Don't like either of them

ii. If the 21g didn't work the 23g didn't work and vice versa

e. No preference, n = 4

9. Did you feel you had adequate training to use the Steripath Device? If No, what would have made the training better?

a. Yes, n = 54

i. Tool just doesn't work for all of Inpatients

ii. The training was good

b. No, n = 10

i. Only geared towards using it

ii. Never told how to fix the problems when the tricks did not work

iii. Better training needed, more hands-on real person before implementing

iv. Trainers were very vague on how to move forward with difficult draws

v. Rushed through the training by Steripath Rep

vi. Trainer not very experienced with collecting blood so unable to understand questions being asked

c. Yes & No, n = 5

i. Would Like to have seen in person/at bedside training before use

ii. Magnolia staff presented well, but did not know Mayo processes so different information shared based on person sharing it

iii. Would have been nice if leadership would accompany staff on floor for first 1 or 2 blood cultures at the start of the pilot

iv. Original training great, would have liked to have a training session again later in the pilot after discovering some of the problems

12. Please describe any difficulties in measuring the volume of blood while filling the bottles you encountered?

a. It's almost impossible to not be crouching over while using the Steripath. Patient beds only go up so far and chairs are even more of an issue

i. Constant bending over to see the volumes creates health risks for taller techs, not all patient beds can be raised

b. ED patient's , limited workspace ED, ICU and gen. care patients. Tubing is too short

i. Pulled the needle out while trying to focus on the volume in the bottle

ii. When patient rooms are not big enough, it is difficult to put the culture bottles vertically and be able to measure the right volume

iii. It was very awkward to use at the bedside because unable to keep bottles vertical

c. The bubbles/foam inside the bottles get tricky once the blood is going in

d. Hard to see. I had to kneel on floor to see the line of blood completely

i. Sometimes it's hard to tilt the bottles upright so you don't know how much is being placed or the tubing is too short for adjustment

ii. Bottles not premarked. A few times I forgot to mark the bottles I remembered as soon as I attached it so I had to watch extra close. Not a very accurate measurement

iii. It is nearly impossible to keep the bottles near eye level to see correctly and prevent over or under filling.

iv. Some bottles already marked but marked incorrectly, being a "notch" or two above or below the actual fill level before drawing

v. You need a flat surface and there is not a flat surface available in certain areas

vi. Contorting body into weird positions

vii. Working around other things (IV poles, food trays, drain containers, etc.) very difficult to get in the correct position to see the volume lines

viii. Bottles marked incorrectly and inconsistency in the broth lines from the manufacturer

e. Patients are often not in ideal positions to have a level place for the specimen

f. Patients moved, rolled over, started struggling or being defensive, combative, etc

g. Working with behavioral patients who moved a lot made this very difficult

h. Hate the whole devices not convenient or accurate in measuring

i. Inconvenient to have to mark the volume levels on the bottles before the collection, especially when the broth in the bottle is at an uneven level to begin with

i. Lining up the volume lines on the bottle with progressive lenses not always easy

ii. Manufacturer labels on the gray bottles not always consistent, sometimes looks like 8mLs other times 11 or 12mLs using the graduation lines on the label

j. You must be level with the bottle to get an accurate volume amount (backaches even with bed raised)
