# Supplementary material for: Comparison of blood culture contamination rates with standard practice versus two blood diversion devices at a single institution
Source: J Clin Microbiol. 2026 Jun 29;64(8):e00085-26. doi: 10.1128/jcm.00085-26 (PMC13463775; doi:10.1128/jcm.00085-26)
Supplement: Supplementary file 5 [file jcm.00085-26-s0006.pdf]

Table S5: Aggregate survey responses from phlebotomists describing their experience with the Steripath with an integrated syringe configuration (Steripath-syringe).

Tabulated Survey Responses:

2. Please describe any difficulties in using the Steripath with the syringe device

a. Activating the waste chamber
- i. It pulled the needle out of a lot of patient's arms
- ii. Activating the waste change is kind of hard
- iii. Suction would collapse more delicate veins no matter how slow it was squeezed
- iv. On smaller veins, when activating the waste chamber, the activation blew the veins
- v. If the blood was flowing slowly, difficult to determine when the waste chamber was full

b. Switching out syringes
- i. Very difficult to pinch off tubing, hold the waste chamber, and twist off the syringe
- ii. No way to stabilize the butterfly when switching out syringes and needle is pulled, falls out of arm
- iii. Hard to change out syringes, especially when the syringe seems to seal to well and is hard to twist off
- iv. Would be better if it was a 30mL syringe
- v. If tubing not pinched off tight enough blood flows out and makes a mess
- vi. Challenging to ensure the clear luer stayed in place so the butterfly did not unscrew
- vii. Transferring a new syringe onto the device is difficult
- viii. Blood stops flowing after switching out syringe

c. Difficult Collections
- i. If close to knuckle, really bulky and difficult to keep the needle from being pulled out
- ii. Suction would collapse more delicate veins no matter how slow
- iii. Think they are fun to use, but for delicate veins, they only cause problems
- iv. Veins were too small to handle the vacuum, to much pressure on small veins
- v. The 2mL waste makes collecting blood difficult because a micro amount is all that you can get from some patients. Sometimes it is hard to take a waste and get any more blood on difficult patients
- vi. Having only the 10mL to start with
- vii. Very difficult to use for patient with small veins, never got blood when used on a hand vein

d. The whole device is very bulky, feels easier to accidently pull the needle out
e. Steripath devices occupy more space in the carts so it is difficult to stock enough for multiple sets of cultures
f. The devices take up more space in the bio buckets, so they fill up faster
g. Have had the blood clot and stop running, even when drawing slowly because of the time it takes to fill the waste chamber
h. Takes longer to complete the collection process
i. Need longer tubing, so needle is not pulled out when switching out syringes
j. Trying to determine the most comfortable way to hold the waste chamber when activating
k. Loose tubing connector (butterfly luer) on the waste chamber. Had to make sure it was tight and connected prior to use
l. Not enough hands to do everything required

5. When unable to use the Steripath with syringe device for certain patients or patient populations, what limited the use of the device?

a. Very small veins, fragile veins, blew the veins
b. Felt the pull of the waste vacuum would collapse the vein
c. The device is too bulky
d. If the patient is a difficult and can barely get blood, could not waste 2mL
e. Knuckle veins or any other less optimal location did not work well
f. Patient jittery and needed to have more control over the collection for patient safety
g. Did not use on surface veins or small veins
h. Vein/skin integrity, limited space access at bedside, due to other services working on patient
i. Hand veins when switching out syringes was extremely difficult
j. Inability to hold everything and get the sample, needed an additional set of hands or tech
k. Combative patients, confused patients, uncooperative patients
l. Busy rooms and working around other services, emergency situations, patient cannot hold still
m. Swollen arms, bruises, poor veins, etc.
n. Patients in critical care or on NPO with limited venous access for venipuncture. Often the vacuum created by the steripath device would rupture a smaller vein
o. No supply available in cart, used them all
p. Arm restrictions and no good veins for a stick, difficult position and not able to hold the needle

6. Did you try to use the Steripath with syringe device for every blood culture order? If No, why not?

a. Tried and failed most of the time, extra pokes for the patient
b. Once veins started to blow, I stopped using them
c. After evaluating the vein, I realized it would not hold up to the pressure, did not use
d. Bulky and cheaply made, prefer using what works best for the patient and myself
e. Sometimes veins are just too delicate
f. Ran out of stock of them due to limited space for them
g. Difficult patient collections, small veins, damaged veins, behavioral patients
h. Cultures and multiple labs, can only collect 3 syringes before needing to ask for help to dispense

i. Did not have the right gauge on cart for the collection
j. Prefer the Steripath vacutainer better
k. If difficult to get the first culture, would not use for the 2nd culture
l. Collections in the hands
m. If the patient was difficult would use the old way to get all the blood possible
n. Poor veins, bruises, difficult patient
o. We seem to have an increased population of difficult sticks, and some you are just hoping to get 1 ml of blood, I felt the steripath syringe was too bulky and risky to have to waste blood when i thought i would only be getting 1 to 2mls out of the vein
p. When professional judgment said the device would damage the only vein available
q. Doesn't work for all patients' veins (difficult sticks, difficult getting to the patient with extra bulky equipment, pressure on the vein when using the system vs a butterfly and small syringes. Not able to take the time to use the kits when the patient room is too busy or in a hurry

7. What gauge did you prefer, 21g or 23g, and Why?
a. 21g, n = 40
    i. Pulls so much better, better flow, less chance of hemolysis
    ii. Patient had good veins
    iii. Never liked the 21g, had no success with 23g
    iv. Provides a better sample
b. 23g, n = 18
v. Most patient veins are not that well
vi. Blood culture patients are usually doing poorly resulting in vasoconstriction
vii. Seems like less issues, more successful
viii. Delicate, small veins
c. Both = 16
ix. Used what was best for the vein
d. Neither = 2
x. Don't like either of them, too hard on veins

9. Did you feel you had adequate training to use the Steripath with syringe device? If No, what would have made the training better?
a. Yes, n = 71
    i. Training was good, My Learning assignment was good, trainers knew what they were doing
b. No, n = 3
    i. Device just showed up, there was no training
    ii. Would be better with at bedside training, situation different than on fake arm
c. N/A, n = 1
d. Maybe, n = 1
